# Editorial for epidemiology and infection, special issue on post-COVID condition

**DOI:** 10.1017/S0950268825100149

**Published:** 2025-12-02

**Authors:** Sharon Saydah

**Affiliations:** National Center for Immunizations and Respiratory Disease, Centers for Disease Control and Prevention, Atlanta, GA, USA

Long COVID presents a unique challenge to the research, medical, and public health communities. The challenge starts with the name used by researchers, clinicians, and patients (Long COVID, Post-COVID Conditions, or Post-acute Sequelae of SARS-CoV-2) and continues with the many symptoms and conditions included in the broad definition that can vary across studies and countries. Long COVID is complex and multifaceted, making it difficult to describe and define. Since Long COVID was first described by patients, the scientific and medical community has learned a great deal [1]. We have a better understanding of groups at increased risk for Long COVID [2]. Estimates on the number of people impacted by Long COVID vary by study population and study design, but recent estimates appear to be coalescing around 3%–5% among adults and 0.5%–2% among children [2–4], an estimated 65 million people globally [5, 6]. While the risk of developing Long COVID is low, the worldwide burden is quite high due to the large number of people who were and continue to be infected with SARS-CoV-2. Epidemiologic research is well poised to address many of the remaining questions surrounding Long COVID through observational studies that examine risk factors and phenotypes, explore potential biological mechanisms, and assess effectiveness of prevention and treatment. Each of the articles in this collection addresses a research gap and may help guide future efforts in unlocking the mysteries of Long COVID. This collection of articles on Long COVID advances the field by addressing many of the key questions and topics through original research, review of the state of the science, and recommendations for a path forward.

One challenge in studying and understanding Long COVID is the need to prospectively follow individuals and monitor symptoms and conditions in the months and years following infection. Three of the articles in this collection do just that [7–9].

Beale and colleagues [7] present findings from Virus Watch, a prospective community cohort in England on the prevalence of Long COVID by SARS-CoV-2 variant period. The good news is that the occurrence of Long COVID following infection has declined since early in the pandemic. Unfortunately, this does not mean that Long COVID no longer occurs. While the numbers of people impacted going forward may decrease, there will still be people who experience long-term symptoms. In addition to longer term symptoms from SARS-CoV-2 infection, this study reports that people experiencing other acute respiratory infections (ARIs) may also experience longer term symptoms. As the authors point out, it will be important to continue to monitor longer term symptoms associated with infection by future SARS-CoV-2 variants and other respiratory viruses.

Austhof and colleagues [8] report on persisting gastrointestinal (GI) symptoms and irritable bowel syndrome (IBS) following SARS-CoV-2 infections among a prospective cohort of adults living in Arizona. Gut immunology and the microbiome disruption are hypothesized to lead to ongoing GI symptoms possibly through viral persistence [10]. GI and IBS are not consistently reported by patients with Long COVID [11, 12] but can cause debilitating symptoms that impact quality of life. By prospectively following participants, Austhof and colleagues [8] are able to capture the ongoing impact and persistence of these symptoms. GI and IBS are symptoms and conditions that can lead to increased health care needs and may lend themselves to specific viral therapeutic targets [10].

One of the many challenges of Long COVID, as mentioned, is its clinical diagnosis and definition. This can lead to multiple treatment approaches to relieve symptoms. The goal of these treatments is typically to improve quality of life and reduce limitations on activities. The best way to measure this is by asking individuals about their symptoms and well-being. The study by Gorelik and colleagues [9] did that by asking adults, both those previously infected with SARS-CoV-2 and not infected, about their general well-being along with ongoing symptoms. They found that non-specific symptoms, such as fatigue, had the largest impact on well-being of participants. Given the broad definition of Long COVID, further refining the definition to include a measure of well-being could help guide clinicians as to what symptoms may require intervention or treatment to improve overall well-being of persons with Long COVID.

In addition to prospective studies, cross-sectional studies can provide additional insights and a snapshot in time. Gerritzen et al. [13] furthers the understanding by describing phenotypes of clusters of symptoms reported by patients with Long COVID. These phenotypes they identified are also tied to health-related quality of life and healthcare use. In general, the more symptoms experienced by patients with Long COVID, the more healthcare use and the poorer health-related quality of life. The articles by Gorelik and Gerritzen highlight the importance of listening to patients with Long COVID and including measures of quality of life or well-being in further studies of Long COVID and in clinical care [9, 13]. The findings also suggest that including a statement on the impact of symptoms on everyday activities, similar to the World Health Organization definition of Post-COVID-19 Condition, could assist in refining the definition and helping guide clinical care [14]. Abucan and colleagues [15] found that health-related quality-of-life measures improve in time since SARS-CoV-2, but many patients continue to experience impairments up to 18 months from infection. This study included a comparison group of non-infected individuals, which adds evidence supporting that the health-related quality-of-life impairments are likely attributed to SARS-CoV-2 infection.

Given the large number of studies in this area, the review articles in this collection [15, 16] assist researchers by synthesizing the research to date and describing overall patterns. The first one by Afrisham and colleagues [16]provides an important review of mechanisms associated with some of the more consequential longer term complications, including renal, cardiac, neurological, cutaneous, and coagulatic manifestations. The review presents the evidence to date on how the SARS-CoV-2 virus may be directly influencing cells and leading to these complications. By developing treatments that target these mechanisms, researchers might be able to prevent the development of these complications. For example, if a patient who may be at higher risk for renal disease is infected with SARS-CoV-2, a targeted treatment may reduce the risk of progression to kidney failure due to the infection.

Along with identifying potential targets for prevention or treatment based on viral pathophysiology, another tool available is COVID-19 vaccination. COVID-19 vaccines are one of our most effective tools [17] to reduce the risk of acute COVID-19, the severity of acute illness, and the risk of Long COVID. Pasculli and colleagues [18] examined differences in Long COVID symptoms and phenotypes among adults who were and were not vaccinated prior to SARS-CoV-2 infection. While there were few differences in Long COVID symptoms across the groups, some phenotypes were more common among the unvaccinated group, highlighting the complex relationship between Long COVID, distinct phenotypes of symptoms, and vaccination status. The review by Jennings et al. [19] provides further evidence that vaccination prior to infection reduces the risk of Long COVID. The review then extends this to provide evidence that vaccination after infection also reduces the occurrence of Long COVID. However, there is no evidence that COVID-19 vaccination reduces or improves symptoms of Long COVID among people who already have Long COVID.

Epidemiologic studies can also provide insights into treatments for people with Long COVID. One potential therapeutic approach to treat some of the more commonly reported and debilitating conditions of Long COVID, neuropsychiatric symptoms and conditions, is proposed by Schwartz and Suskind [20]. A recent meta-analysis found that one in five people with Long COVID experienced these symptoms and conditions [21]. Clinical trials are one path forward to identifying effective treatments amid the clinical uncertainty of Long COVID.

We still have a great deal to learn and understand from the underlying biological mechanisms, to whether there are distinct phenotypes, and how each of these factors may ultimately guide treatment and possible prevention. Epidemiological studies have been at the forefront and are a key component to moving this field forward. While the COVID-19 public health emergency has ended, SARS-CoV-2 is going to continue to circulate, along with other respiratory viruses, throughout the world, and Long COVID will continue to occur. A goal of Long COVID research and the public health response is to reduce the occurrence of Long COVID and improve the lives of those who are experiencing Long COVID. Epidemiologic studies, from cross sectional to prospective as well as to clinical trials, will play crucial roles in unlocking the mysteries of Long COVID.
